# Measurement Noise Model for Depth Camera-Based People Tracking

**DOI:** 10.3390/s21134488

**Published:** 2021-06-30

**Authors:** Otto Korkalo, Tapio Takala

**Affiliations:** 1 VTT Technical Research Centre of Finland Ltd., P.O. Box 1000, FI-02044 Espoo, Finland; 2 Department of Computer Science, Aalto University, FI-00076 Espoo, Finland; Tapio.Takala@aalto.fi

**Keywords:** people tracking, depth cameras, measurement noise models, data fusion, multiple-view tracking

## Abstract

Depth cameras are widely used in people tracking applications. They typically suffer from significant range measurement noise, which causes uncertainty in the detections made of the people. The data fusion, state estimation and data association tasks require that the measurement uncertainty is modelled, especially in multi-sensor systems. Measurement noise models for different kinds of depth sensors have been proposed, however, the existing approaches require manual calibration procedures which can be impractical to conduct in real-life scenarios. In this paper, we present a new measurement noise model for depth camera-based people tracking. In our tracking solution, we utilise the so-called plan-view approach, where the 3D measurements are transformed to the floor plane, and the tracking problem is solved in 2D. We directly model the measurement noise in the plan-view domain, and the errors that originate from the imaging process and the geometric transformations of the 3D data are combined. We also present a method for directly defining the noise models from the observations. Together with our depth sensor network self-calibration routine, the approach allows fast and practical deployment of depth-based people tracking systems.

## 1. Introduction

Computer vision-based people tracking has many applications from surveillance to people flow analysis, smart environments and human–computer interaction. The typical vision-based people tracking system consists of several components such as people detection and appearance modelling, data fusion and tracking, trajectory and behaviour analysis, and sensor and system calibration. In recent decades, different sub-problems have been addressed in numerous publications, where the main focus has been on methods using monochromatic and RGB cameras. Other types of camera sensors such as depth cameras have also been used, and the early depth-based people tracking methods have relied on stereo disparity [1] and Time-of-Flight (ToF) cameras [2].

Depth sensors have many benefits compared to RGB cameras in people tracking. They are privacy preserving and the person’s identity is nearly impossible to recognise from the depth data by the human eye. ToF and structured light sensors are based on active illumination, and they can also be operated in complete darkness. Depth sensors directly capture the scene in 2.5D, and less geometrical assumptions (e.g., ground plane hypothesis) are needed for people localisation and tracking. For the people tracking, the output of the depth cameras directly provides features on the metric coordinate system.

Surveillance, smart environments and people flow analysis are examples of people tracking applications where a typical camera set-up consists of a network of multiple, possibly heterogeneous camera sensors. Multi-sensor systems are used to extend the tracking area, to prevent occlusions and to produce more accurate state estimates for the trajectories. In such systems, the data fusion plays a crucial role. The observations have to be mapped to the common coordinate frame for positioning and tracking and the uncertainties of the measurements are used for optimal state estimation and to limit the search space in data association step.

Depth cameras suffer from depth measurement noise, which may depend on various factors such as environment’s lighting conditions and the scene geometry and materials being observed [3,4,5]. The measurement noise typically varies across the depth image pixels, but the most affecting factor is the measured distance. Typically structured light (SL) sensors suffer more on the range-dependent measurement noise compared to ToF [3,6]. Specifically, with Microsoft Kinect SL cameras and similar, depth quantization plays an important role. In a study by Smisek et al., the 1^st^ generation Kinect was analysed, and they reported the depth quantization of approximately 400 mm at the distance of 12 m [7]. Khoshelham et al. reported random errors of 40 mm at a 5 m distance, and the depth resolution of 70 mm at 5 m, and both errors increasing quadratically with respect to the range [4]. Zennaro et al. [6] evaluated and compared the performance of Kinect SL and ToF devices for different applications including people tracking. Due to the higher precision of the captured point clouds, their experiments with the people tracking approach of [8] resulted in 20% better tracking accuracy with ToF sensors.

Depth measurement noise does not only depend on the distance to the measured target, but also on the pixel position on the depth image. In a study by Belhedi et al. [9], the depth measurement errors of ToF cameras were treated as normally distributed, and a 3D thin plate spline (TPS) was used for modelling the standard deviation of the range measurements across the depth image plane. In addition to the noise model, they presented how the measurement errors of each depth image pixel are propagated to 3D, and how this could be incorporated into 3D computer vision applications such as the size measurements of objects. Nguyen et al. [5] developed a noise model for Kinect SL where axial and lateral components are modelled separately, and which are functions of the measured range and the angle between the measured surface and the optical axis of the sensor. Previous methods require manual work determining the parameters for the measurement noise models.

For depth camera-based people tracking applications, it is common to install the sensors to top-view position, or project the captured 3D point clouds to the floor coordinate system, and perform the tracking on so-called plan-view domain. Harville was one of the first to introduce plan-view-based people tracking [1]. He used stereo cameras for the depth imaging, and a Kalman Filter (KF)-based approach for the tracking. Harville also pointed out that the sensor’s depth measurement noise increases proportionally to the range. Stahlschmidt [10] et al. used a ToF camera in the top-view position, and they searched for persons’ heads by finding the local maxima from the captured point clouds. For the tracking, they used a KF-based approach with the Mahalanobis distance-based gating of measurements and a global nearest neighbour data association. In [11], Muscoloni et al. introduced an FPGA implementation of an embedded people tracking node using the plan-view method. The tracking part was implemented by using a combination of KF and the mean-shift algorithm. Liu et al. [12] presented an RGB-D camera-based people detection and tracking approach that operates in the plan-view domain, and where the combination of colour and spatial information was used to describe the appearance of the tracks. In their solution, a KF-based tracker was used to predict the positions of the tracks for reducing the search space of the data association. Almazan et al. [13] also used a combination of KF and the mean shift algorithm for tracking across multiple non-overlapping depth sensors in the plan-view domain. The predictions of the uncertainties of the targets’ positions were used to narrow down the search space of the people detector.

The plan-view approach scales naturally to multiple camera systems. Crombrugge et al. [14] transformed the point clouds from different sensors to the common plan-view coordinate frame, and created a fused 3D point density map from which people were detected. For the tracking, they used KF with a global data association scheme. They also conducted experiments on how a sensor’s installation height, angle, number of the sensors and the depth measurement noise affected the tracking results. However, they did not address the range dependent noise. Tseng et al. presented a similar approach, and they fused the input point clouds from different sensors before performing the head detection step [15]. The approach of Munaro et al. [16] supports multiple types of depth cameras for multiple sensor-based people tracking. They track the people in the floor plane coordinate system, and utilize the unscented Kalman filter and constant velocity state space model for state estimation. Although Munaro et al. use the Mahalanobis distances between the predicted track positions and observations for gating and data association tasks, they did not report details about the measurement noise models used in their approach.

Multiple-view depth camera systems have been used for skeleton tracking as well. Zhang et al. [17] fused the point clouds from different depth cameras, and used a particle filter-based method for estimating the pose of the skeleton. They used a signed distance function for the goodness-of-fit measure, and assumed a zero-mean Gaussian measurement noise. Carraro et al. [18] also fused the point clouds, but they detected the skeletons from synthetic depth images rendered from the frontal view of the person. For the registration of the point clouds, they used the iterative closest point algorithm (ICP). They argued that the ICP is particularly effective for the sensors that have significant errors in depth estimation, but they did not address the noise model itself.

Although the state estimation, the data association and the gating steps of any depth camera-based people tracking system benefit from an accurate depth measurement noise model, previous studies do not address the problem. On the other hand, the existing depth camera noise models require a manual calibration step to be carried out before use. In many practical people tracking applications, it would be beneficial to avoid any manual calibration steps for the fast deployment of the systems. In this paper, we focus on determining the uncertainty of the measurements in plan-view-based people tracking systems. The main contributions of this paper are:

1. A non-parametric, state-dependent measurement noise model for depth camera-based people tracking systems that utilise the plan-view approach. In our model, the measurement noise is modelled in the plan-view domain as 2D Gaussian distribution. The measurements are expressed in the polar coordinate system, and the error distributions for the bearing and range are separately defined as deviations from predefined reference points;
2. A method for determining the noise model directly from the people detections. For reference, we use either the ground truth points or the trajectories that are reconstructed in our depth camera network autocalibration method.

Our approach has the following benefits:

- As we do not have any explicit model for the measurement noise, the approach can be used with different kinds of depth cameras, and even with depth sensor networks that consist of a heterogeneous set of sensors.
- The noise model can be directly derived from people detections, and no separate calibration routines or calibration targets (such as checkerboard planes) are needed. When used in conjunction with our depth camera network self-calibration routine [19], it allows fully automatic system configuration.

The rest of this paper is as follows. In Section 2, we describe our people tracking system, and we present the context for the proposed measurement noise model. The noise model is detailed in Section 3, and the experiments for evaluating it are described in Section 4. The results of the experiments are presented in Section 5, and the discussion and conclusion are in Section 6.

## 2. People Tracking with Depth Sensor Network

### 2.1. Overview

Our people tracking solution is based on a centralised tracker architecture where each camera is responsible for independently providing observations from people. The observations are sent to the server, which is responsible for data association and tracking. For the plan-view-based people tracking, the persons may be detected from the depth maps directly and then transformed to the plan-view coordinate system. The other approach is to first transform the raw input data to the plan-view domain, and detect the persons from the result. We used the latter approach. The first step in the processing was to transform the input point clouds to the plan-view coordinate system. From the transformed point clouds, we construct the height maps by rendering the point clouds from the top-view perspective using an orthogonal projection so that the topmost pixel value is saved. We detect the persons from the height maps by utilising background subtraction, and searching for the local maxima from the foreground pixels. The detections are then transmitted to the tracking server, which transforms them to the global coordinate system, and performs the tracking.

### 2.2. Plan-View Transformation

For each camera node, we construct the height map as follows. We first transform the 2.5D depth maps captured from the camera to point clouds using the known intrinsic calibration. It should be noted that as we use a non-linear mapping (see Section 2.4) for transforming the observations from different sensors to the global coordinate system, the intrinsics does not have to be accurate, and the lens distortions can be ignored at this point. Let us denote a homogeneous depth image pixel at $u={\left(u,v\right)}^{⊤}$ with UD=(d·u,d·v,d,1)⊤, where *d* is the measured distance from the camera to the object. Then, the transformation of the pixel to the top-view height map image pixel UH can be written as

(1) UH=PRK−1UD

where K is the 4×4 sensor intrinsic parameter matrix, R is a 4×4 rotation matrix that transforms the 3D point cloud so that the floor plane becomes parallel to the sensor image plane, and P is a 4×4 orthogonal projection matrix that scales the points to predefined height map image size:

(2) $$P=[\begin{array}{cccc} \frac{s}{\Delta y} & 0 & 0 & -\frac{sx_{0}}{\Delta y}\\ 0 & \frac{s}{\Delta y} & 0 & -\frac{sy_{0}}{\Delta y}\\ 0 & 0 & -1 & h\\ 0 & 0 & 0 & 1 \end{array}]$$

where *s* is the height map image size in pixels, $x_{0}$ and $y_{0}$ are the minimum *x* and *y* values of the top-view point cloud, and Δx and Δy are the ranges of the point cloud in *x* and *y* dimensions. As mentioned previously, *x* and *y* dimensions are scaled with the same scale factor, and the transformation corresponds to a square height map image.

### 2.3. Tracking

For the tracking, we used a standard Kalman filter (KF) state estimation. We assumed that the changes in the target velocity are small, and we used a (nearly) constant velocity (CV) dynamic model. In KF-based tracking, the system process noise covariance Q defines how fast the velocity is allowed to change. In our tracker, we used the following approximation for the system process noise covariance [20]:

(3) $$Q=q[\begin{array}{cccc} \frac{1}{3}\Delta t^{3} & \frac{1}{2}\Delta t^{2} & 0 & 0\\ \frac{1}{2}\Delta t^{2} & \Delta t & 0 & 0\\ 0 & 0 & \frac{1}{3}\Delta t^{3} & \frac{1}{2}\Delta t^{2}\\ 0 & 0 & \frac{1}{2}\Delta t^{2} & \Delta t \end{array}],$$

where Δt is the sampling period, and the magnitude of the process noise is defined by the design parameter *q*.

Denoting a track with x at a timestep *k*, we first predict a new state estimate ${\hat{x}}_{k|k-1}$, and propagate the uncertainty of the state $P_{k|k-1}$ using the standard Kalman filter predict equations. Denoting the observations with z, we calculate the squared Mahalanobis distance for each track-observation pair with:

(4) $$D={\tilde{y}}_{k}^{⊤}S_{k}^{-1}{\tilde{y}}_{k}$$

where ${\tilde{y}}_{k}$ is the innovation:

(5) $${\tilde{y}}_{k}=z_{k}-H{\hat{x}}_{k|k-1},$$

and $S_{k}$ is the corresponding innovation covariance:

(6) $$S_{k}=R_{k}+HP_{k|k-1}H^{⊤}.$$

H denotes the observation model matrix and $R_{k}$ is the noise covariance matrix that corresponds to $z_{k}$. In our state-dependent measurement noise model, the measurement noise covariance depends on the target’s distance and angle relative to the sensor, and it is updated whenever new observations are delivered from the sensors. The Mahalanobis distances *D* are used to compute the cost for each track-measurement pair in the data association step, and we solve the global optimum for the best pairs using the Hungarian algorithm. Furthermore, the distances *D* can be used for gating and ignoring unlikely match candidates. The states of the tracks are then updated using the standard Kalman filter update equations. For this purpose, one needs to compute the Kalman gain, which involves using $S_{k}$ and it weighs the importance of the observations based on their uncertainty.

For track management, we used the following approach. The observations that are not associated to any of the existing tracks initiate new tracks which are labelled as tentative. The tentative tracks are updated similarly to the existing tracks, and as they are being successfully updated for a predefined time interval, their status is set to confirmed. The confirmed tracks that are not associated to any of the observations (due to occlusions, noise, or a target moving out of the cameras’ view) are labelled as lost. The lost tracks’ states and corresponding uncertainties are being updated without Kalman filter’s correction step until an observation can be associated to the track again. If no observations are matched for a predefined time interval, the track is deleted.

### 2.4. Transforming Observations to Global Frame

For the data fusion and tracking, the observations from different sensors are transformed from the local camera plan-view coordinate frame to the global plan-view frame. In an ideal case, 2D rigid transformations with three degrees-of-freedom (position and direction angle) accurately align the observations, but inaccurate plan-view transformation parameters, imprecise intrinsic calibration of the sensors, and the depth measurement distortions induce linear and non-linear errors that cannot be modelled with the rigid transformations only. We applied the thin-plate-spline (TPS) mappings [21] for both transforming the observations to the global frame, and for directly compensating the errors in the plan-view domain. The details of the approach are presented in [19], however, we summarize the main points here. The TPS mappings have been widely used in many computer vision applications, such as point set matching and image warping, and they have also been applied for modelling depth camera distortions [22]. The TPS is an $R^{2}\to R$ mapping that has an affine part $M_{A}$ and a non-linear part defined by a set of *p* control points $c_{i}$, TPS kernel ϕ and associated weights. For $R^{2}\to R^{2}$ TPS, two mappings that share common control points are used. Denoting the 2D observations in the local camera plan-view coordinate system with xC, and the transformed points in the global coordinate system with xG, the mapping can be expressed with:

(7) xG=MAxC+WΦ(xC),

where W is a 3×p matrix of column vectors $w_{i}={\left(w_{xi},w_{yi},0\right)}^{⊤}$ and $w_{xi}$ and $w_{yi}$ are the weights in the *x* and *y* dimensions, and $\Phi \left(x\right)={\left(\phi_{1}\left(x\right),\phi_{2}\left(x\right),\cdots ,\phi_{p}\left(x\right)\right)}^{⊤}$. The kernel $\phi_{i}\left(x\right)$ is defined as $\phi_{i}\left(x\right)=d{\left(x,c_{i}\right)}^{2}\log\left(d\left(x,c_{i}\right)\right)$, where $d=\left|\right|\left(c_{xi},c_{yi}\right)-\left(x,y\right)\left|\right|$.

### 2.5. Camera Network Calibration

The parameters of the TPS mappings are determined by using the camera observations in the local plan-view domain and corresponding reference points of the global coordinate system. The reference points can be collected, e.g., with a self-navigating mobile robot, which knows its exact location at every time step. To avoid manual tasks in the calibration process, we presented an autocalibration method in [19]. The goal of autocalibrating is two-fold: first, we determine the optimal structure of the reference points, and second, using the reference points, we can compute the TPS mappings for each camera, as well as the measurement noise models as described in Section 3. In the following, we briefly revise the method.

We first compute initial pairwise rigid transformations for each sensor pair. The transformations map the observations from the first camera’s plan-view coordinate system to the second camera’s plan-view coordinate system. For finding the mappings, we use a temporal matching of the observations and an RANSAC-based approach. A specific score is used to determine the convergence of the RANSAC, and for identifying the sensor pairs that share the common view. One camera is selected as a base camera whose coordinate system is used as a global frame. Then, using the initial pairwise mappings, we compute the transformation chains from the base camera to the rest of the cameras. The inlier points from the RANSAC scheme are used as initial reference points, and their positions in global frame are computed by transforming them with the transformation chains and averaging the results.

We apply a global optimisation routine similar to the 3D bundle adjustment for optimising the reference point positions and the rigid mapping parameters. Denoting the (homogeneous) 2D reference points in the global frame with x^iG, and the corresponding observations delivered by the sensor *j* with xCij, the solution is obtained by solving the following minimization problem:

(8) arg minMR,x^G{∑i=1n∑j=1mv(i,j)ρd(x^iG,MRjxCij)2}

where $M_{R}^{j}$ is the rigid transformation of the *j*th sensor and v(i,j) is an indicator function that has a value of 1 if the point *i* was measured by the *j*th sensor, and 0 otherwise. The measurements xCij are the inliers resulting from the RANSAC method. Finally, using the original sensor observations and the optimized reference points, we compute the TPS mappings for each camera.

## 3. Measurement Noise Model for Plan-View Tracking

### 3.1. Error Sources of Depth Sensors

According to various studies [3,4,5], depth cameras suffer from multiple types of imaging errors. Lens distortion, inaccurate intrinsic calibration, image pixel and depth noise, depth quantization and sensor-specific imaging processes cause systematic and random errors to the depth images. Due to active illumination, the devices suffer from temperature drift which may have a significant effect on depth estimation. Both structured light and time-of-flight type of depth cameras use active illumination and emit near-infrared light (NIR). Thus, the sunlight and other bright light sources emitting similar wavelengths to NIR may disturb or even completely prevent the depth estimation process.

Similarly, in multi-sensor set-ups, the emitted light from one sensor may interfere with another sensor and cause undesired results such as non-dense depth maps and flickering depth pixels. The third source for the errors depends on the targets being observed. ToF cameras suffer from depth estimation biases for low NIR reflectivity targets, which may cause problems for tracking persons wearing certain types of clothes. Semi-transparent and scattering materials do not perfectly reflect the light, and the depth measurement errors are likely to occur with such objects. Highly reflective surfaces may induce multi-path effects, where the emitted light does not travel directly from the target to the sensor. Such effects lead to wrong distance estimation, and part of the scene may appear at an incorrect distance.

We aimed to model the systematic pixel and depth estimation noise of the depth cameras. We did not take into account the random deviations that may occur due to the multi-path effects, changing imaging conditions, flickering that occurs from low reflectivity targets, or other phenomena that may change due to the imaging set-up. Furthermore, it was assumed that the objects being observed did not have significant difference in their depth response. However, we did not assume explicit function for the systematic measurement noise. Instead, we used lookup-tables for modelling the measurement noise. Thus, the model can be used with different kinds of depth cameras and detection algorithms, as long as the measurement noise remains stationary.

### 3.2. Measurement Noise Model

The depth image pixels UD are transformed to the height map images in the local camera coordinate system using Equation (1). We detected the people from the height maps, and transformed the observations back to the metric plan-view coordinate system. These observations are then transformed to the global plan-view coordinate frame by the TPS mappings defined in Equation (7). Formally, the measurement error covariance matrices of the depth image pixels ΣUD are propagated to the global plan-view coordinate system as follows:

(9) ΣxG=JT1ΣUDJT1⊤,

where $T_{1}$ is the combined transformation from the depth image domain to the global coordinate frame, and $J_{T_{1}}$ is the Jacobian of $T_{1}$.

Similarly to [5], we model the measurement noise for each camera in camera-centric polar coordinate system. We first transform the measurements xG and the corresponding reference points x^G to the local plan-view domain of the camera, and convert them to the polar coordinates. The uncertainty of a point is then defined by a 2×2 error covariance matrix:

(10) ΣpC=σθ200σr2,

where pC denotes a point in the polar system, $\sigma_{\theta }^{2}$ is the variance of the direction angle and $\sigma_{r}^{2}$ is the variance of the observation’s distance from the sensor. In our model, $\sigma_{\theta }^{2}$ and $\sigma_{r}^{2}$ are both functions of θ and *r*:

(11) $$\sigma_{\theta }^{2}=f_{\theta }\left(\theta ,r\right)$$

and:

(12) $$\sigma_{r}^{2}=f_{r}\left(\theta ,r\right).$$

The measurement error covariance matrices can be then transformed to the Cartesian system with:

(13) ΣxC=JT2ΣpCJT2⊤,

where $T_{2}$ is the polar to Cartesian transformation, and $J_{T_{2}}$ is the corresponding Jacobian matrix. ΣxC can be propagated to the global coordinate frame in a similar manner.

Since the errors in direction θ and range *r* are almost independent, the error covariance matrix becomes (nearly) diagonal in camera-centric polar coordinate system. Thus, we only need to estimate two functions to model the measurement noise. We did not make any assumptions about $f_{\theta }$ or $f_{r}$. Instead, we created two 2D variance maps, one for $\sigma_{\theta }^{2}$ and one for $\sigma_{r}^{2}$, which contain the variances in different parts of the sensor’s view in the camera’s plan-view coordinate system. In practice, the maps are defined as lookup-tables which are indexed by the values of θ and *r*, and where each element stores the corresponding variance value.

We estimated the values of the lookup tables by computing the average squared deviation of the measurements from the corresponding reference points at each location. As our goal was to directly compute the measurement noise model from the observations of people, we cannot ensure that the points are collected from stationary objects, or that a sufficient amount of measurements are collected from every part of the sensor’s view. Thus, we allocate the observations to spatial bins of predefined size (e.g., 2 degrees × 500 mm), and compute the variance in those bins. To filter out noise and to fill in the bins with no data, we used the Barnes interpolation method, which is designed to work with sparse and unevenly distributed data points.

## 4. Experiments

### 4.1. Experiment Set-Up

For the evaluation of the approach, we created a test area with four depth cameras installed at the corners. The installation height of the cameras was 2.5 m, and the pitch angle was 30 degrees downwards. The dimensions of the test area were approximately 8×8 m, and the cameras were set at the corners of a grid of an approximate size of 10×10 m. The cameras were oriented to maximise their coverage of the test area. We used Asus Xtion PRO depth cameras in our experiments with the resolution of 640×480 pixels and the frame rate of 30 Hz. For operating the depth sensors, we used Raspberry Pi single board PCs that were time synchronised using the network time protocol (NTP).

**Figure 1:**
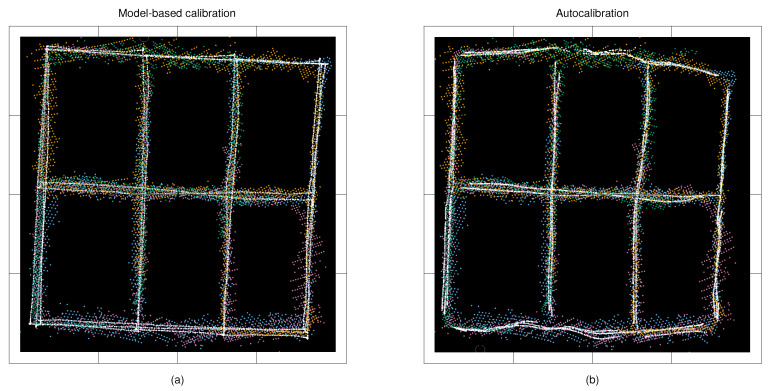
Coloured dots refer to the observations from four different depth cameras, and the white dots refer to the reference points used to perform the geometric calibration, and computing the measurement noise models: (**a**) calibration results from the model-based calibration using the mobile robot’s route as the reference; and (**b**) results from the autocalibration method with the reconstructed route. The tic marks are at two-metre intervals.

We experimented with the approach by using both remote controlled mobile robot and a human as tracking targets. The benefit of using a mobile robot is that it provides a controlled test scenario, and its self localization systems provide accurate ground truth position data. The human experiments better reflect the applicability of the method in real-life use. To mimic a human head, a Styrofoam ball with a diameter of 40 cm was installed on the robot at a height of 150 cm. We drove the robot at a constant speed of 0.5 m/s. The position of the robot in the global plan-view frame was determined by using the wheel odometry, Hokyo UTM-30LX scanning range finder and the adaptive Monte Carlo localisation method as implemented in [23]. The robot localization accuracy was that of a few centimetres, however, the movement caused the Styrofoam ball to randomly wobble at the magnitude of approximately five centimetres. The robot and the person followed the same route in the test area. Figure 1 shows the robot-generated ground truth trajectory, the reconstructed trajectory and the corresponding measurements from different cameras.

### 4.2. Detecting Moving Targets

For detecting moving targets from the depth images, we used a simple background subtraction technique. We first transformed the raw depth maps to height maps in cameras’ local plan-view coordinate system using the method presented in Section 2.2. To model the background for each height map pixel, we maintained long-term and short-term circular buffers of five elements. The short-term buffer was updated at each frame. As a predefined time interval (10 s in our experiments) has elapsed, we updated the long-term buffer with the maximum value of the corresponding short-term buffer. We subtracted the background model from the input height map, and to prevent noise, we placed a threshold on the results using a predefined margin (300 mm in our experiments). For the detection, we used a local maxima search with a search radius of 40 cm. Furthermore, to remove speckle noise from the resulting foreground height map images, we applied a 2D median filter with the kernel size of 5×5 pixels. As the goal was to investigate the measurement noise, not the reliability of the detection method, we manually removed coarse outliers from the observations.

### 4.3. Computing and Evaluating the Measurement Noise

We evaluated the proposed measurement noise model in three use-cases. In the first use-case, we used the mobile robot as a tracking target, and we calibrated the cameras with the TPS mappings using the ground truth robot trajectories as the reference points. In the second use-case, we applied our autocalibration method, and we used the reconstructed robot trajectories as the reference. In the third use-case, we repeated the autocalibration procedure with measurements collected from a person moving along the same route as the robot.

The sensors used in the experiments produce highly distorted data, which we compensate with the TPS mappings. In the autocalibration case, this approach accurately aligns the measurements to the common coordinate system, however, the non-linear errors persist in the reconstructed trajectories. To make the results comparable with the model-based calibration approach, we searched for the TSP mapping between the robot-generated route and the reconstructed route from the autocalibration method, and transformed the measurements using this mapping as well. Figure 1 shows the observations from different sensors, and the corresponding reference points in robot-based experiments.

The variance maps were estimated as described in Section 3.2. Both the robot and the person moved along the straight lines between the grid points so that each segment was traversed at least twice. As the grid points were used to collect the data for computing the true measurement noise, we removed measurements from those areas from the robot data. For the binning of the measurement values, we used a grid of 2 deg × 500 mm. We estimated the variance maps from the data of the moving target, and we could not assume that all the bins have enough data points for computing the variance. Thus, we searched for the bounding polygon of the populated bins to determine the area for which we interpolate the values. For the Barnes interpolation method, we used an implementation of [24]. The method has three parameters, which we selected manually: 10.0 and 5.0 for the smoothing length scales in the angle and range dimensions, and 3 for the number of iterations.

**Figure 2:**
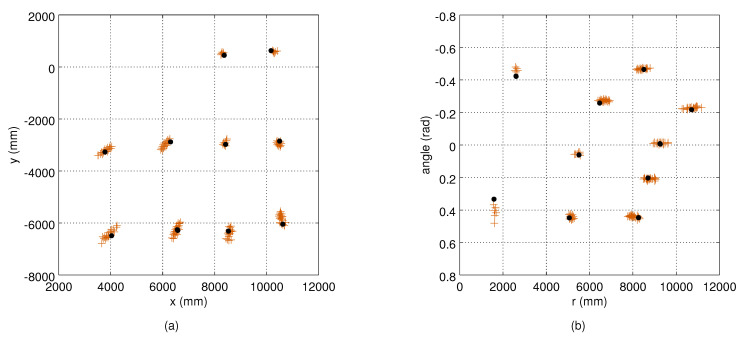
The variation of the observations (red dots) of a static robot (black dots) from Camera 1: (**a**) results in the Cartesian frame; and (**b**) corresponding points transformed to the camera-centric polar coordinate frame. The location and orientation of the camera as in Figure 5.

**Figure 3:**
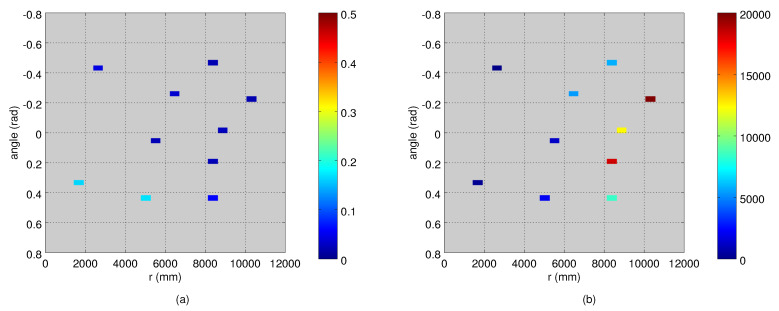
True variances of the observations computed from the data points shown in Figure 2: (**a**) the angle variance ($\deg^{2}$); and (**b**) the range variance (${\mathrm{mm}}^{2}$). The values are computed in 2 deg × 500 mm bins.

For the evaluation, we measured the true measurement noise distributions by collecting approximately 10 s observation sets from stationary targets. The measurement sets were collected from the 12 grid points (see Figure 1) so that between 7 and 10 positions were covered by each camera. Figure 2 illustrates the robot measurements from Camera 1, and the corresponding variances are shown in Figure 3. We used two metrics to compare the true measurement noise covariance and the estimated measurement noise covariance matrices. As an error metric, we used the Kullback–Leibler (KL) divergence from Octave’s mvn package [25]. As a similarity metric, we used the cross-correlation of their density functions, defined as the normalized inner product:

(14) $$R\left(a,b\right)=\frac{\int ab}{\sqrt{\int a^{2}\times \int b^{2}}}.$$

This can be calculated from the error covariance matrices $\Sigma_{a}$ and $\Sigma_{b}$ using the formula:

(15) $$R=2\times \sqrt{\frac{\lambda_{p1}\lambda_{p2}}{\sqrt{\lambda_{a1}\lambda_{a2}\times \lambda_{b1}\lambda_{b2}}}},$$

where $\lambda_{a1,2}$ and $\lambda_{b1,2}$ are eigenvalues of the corresponding matrices, and $\lambda_{p1,2}$ are those of their product distribution, the covariance matrix of which we obtain by

(16) $$\Sigma_{p}=\Sigma_{a}{\left(\Sigma_{a}+\Sigma_{b}\right)}^{-1}\Sigma_{b}.$$

A common approach to model the measurement noise in Kalman filter-based people trackers is to use zero-mean Gaussian noise with predetermined variance, which does not depend on the camera’s orientation or the target’s location relative to the camera. We compared our approach to the static noise model using the standard deviation values of σ= 10 mm, σ= 50 mm and σ= 100 mm.

### 4.4. Tracking

**Figure 4:**
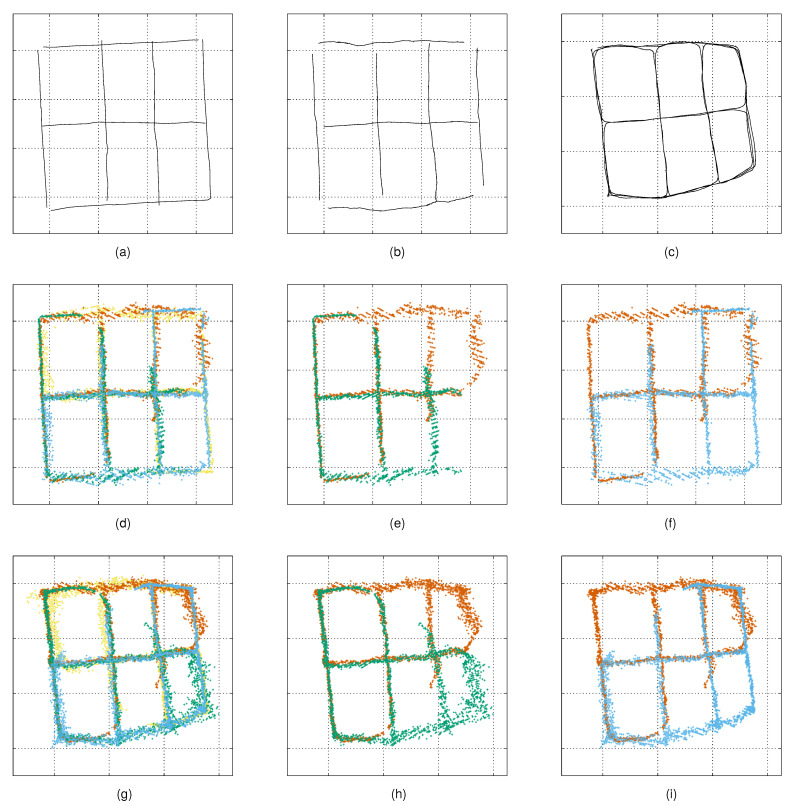
Reference trajectories and measurements used in the tracking experiments—Top-row: (**a**) robot route (ground truth) used in model-based calibration; (**b**) robot route obtained from the autocalibration method aligned with the ground truth; and (**c**) person’s route obtained from the autocalibration method. Middle row: (**d**–**f**) measurements of the robot used in tracking experiments 1–3. Bottom row: (**g**–**i**) measurements of the person used in tracking experiments 1–3.

For experimenting on the proposed noise model with our tracking solution, we conducted experiments with three different camera configurations (tracking experiments 1–3). We computed the variance maps as described in Section 4.3, and we used the same data for the tracking experiments, as follows. For experiment 1, we used the observations from all four cameras, and for experiments 2 and 3, we used the data from two cameras only. Each experiment was conducted using the observations captured from both the mobile robot and the person. To experiment on the performance of the tracker with targets moving at a constant speed and relatively straight paths, we removed all the duplicate segments and turning points from the robot-based data. Figure 4 illustrates the measurements and the corresponding reference trajectories. For each experiment, we applied our measurement noise model with the model-based calibration (for the robot experiments) and the autocalibration methods. We compared the results to the constant measurement noise models with σ=10, σ=50 and σ=100.

The *q* value of Equation (3) defines the magnitude of the internal uncertainty of the state vector of the tracker. In practice, using a small value of *q*, we did not allow rapid changes in the velocity, and the target was assumed to move along smooth curves in space. With a high value of *q*, the uncertainty of the state is assumed higher, and the track is allowed to perform more rapid manoeuvres. Human movement does not necessarily follow the constant velocity model, and the right value for *q* depends on the situation. In our experiments, we used the values q=10, q=100 and q=1000.

We used the root mean squared error (RMSE) values between the estimated target positions and the corresponding reference point positions as an error metric for the accuracy.

## 5. Results

### 5.1. Measurement Noise Covariances

**Figure 5:**
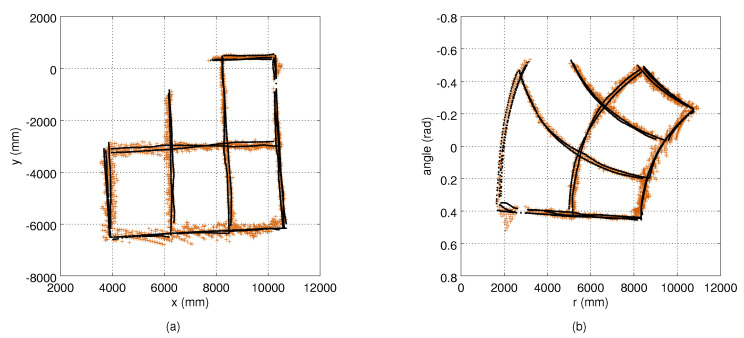
Measurements from Camera 1 (red dots), and the corresponding ground truth (robot’s position, black dots): (**a**) the measurements in the Cartesian frame; (**b**) the measurements transformed to the polar coordinate system of the camera. The location of the camera in (**a**) is at the top right corner, and the viewing direction towards the bottom left. In (**b**), the camera is at the origin with the optical axis along the r dimension.

**Figure 6:**
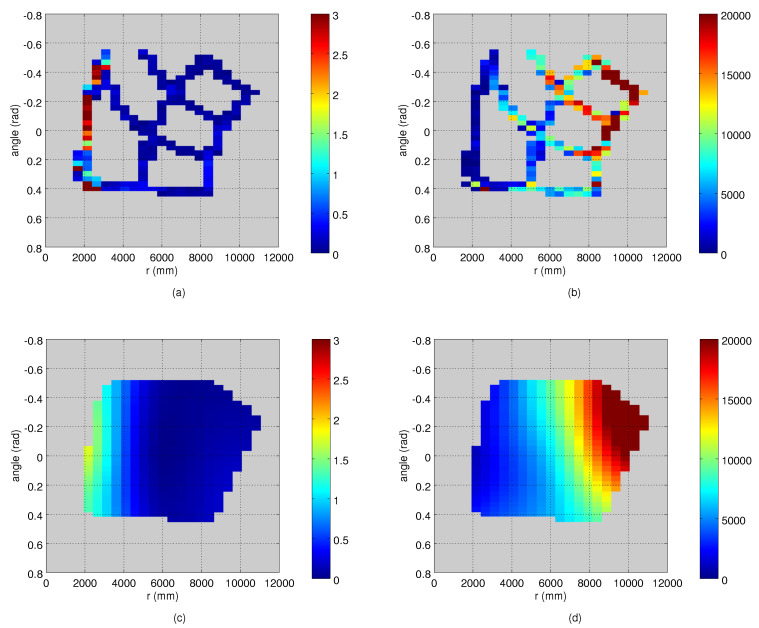
The process for computing the variance maps: (**a**) the variance of the detection angle ($\deg^{2}$) in different bins along the route of a moving target; (**b**) the variance of the range (${\mathrm{mm}}^{2}$) measurements; (**c**,**d**) the variance maps after the interpolation step. The observations are transformed to the plan-view coordinate system of the camera with the origin on the left. The bin size is set to 2 deg × 500 mm.

An example of a robot trajectory and the corresponding measurements from Camera 1 are illustrated in Figure 5, which shows that the measurement noise depends on the target’s distance to the camera. The depth quantization effect is also visible. An example of the estimated variance maps is presented in Figure 6. The original variance values computed at the bins have lots of variation and missing data, and the Barnes interpolation method effectively fills in and smooths out the final variance maps. The true measurement noise measured from the static robot is shown in Figure 2, where the deviation of the measurements in an angular dimension is visible as well. The ground truth variance maps computed from the robot locations of Figure 2 are shown in Figure 3.

**Figure 7:**
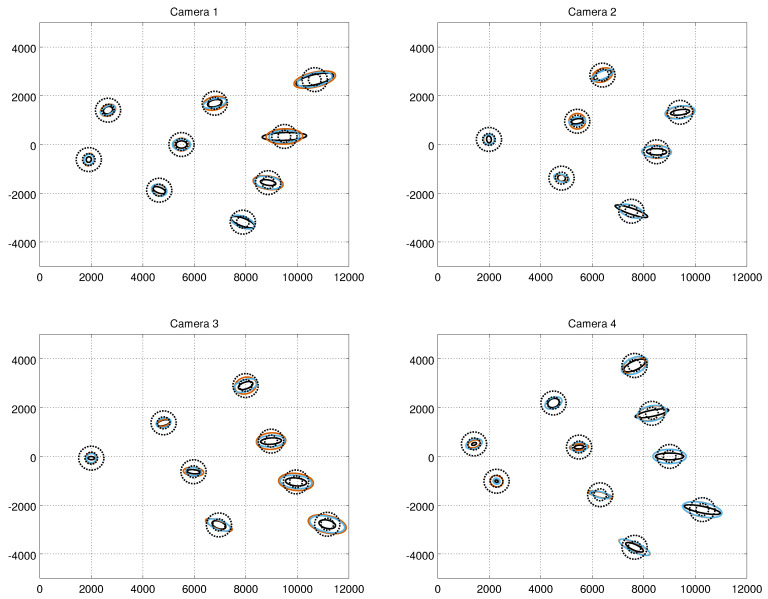
Measurement error ellipses at the confidence level of 95% computed from the robot experiments. The solid black ellipses refer to the ground truth, the smaller dashed black circles to the constant noise model with σ = 10, the larger dashed ellipses to the constant noise model with σ = 50, the red ellipses to the results obtained with the model-based calibration, and the blue ellipses to the results obtained with the autocalibration method. The error ellipses are shown in the camera-centric Cartesian plan-view coordinate system.

**Figure 8:**
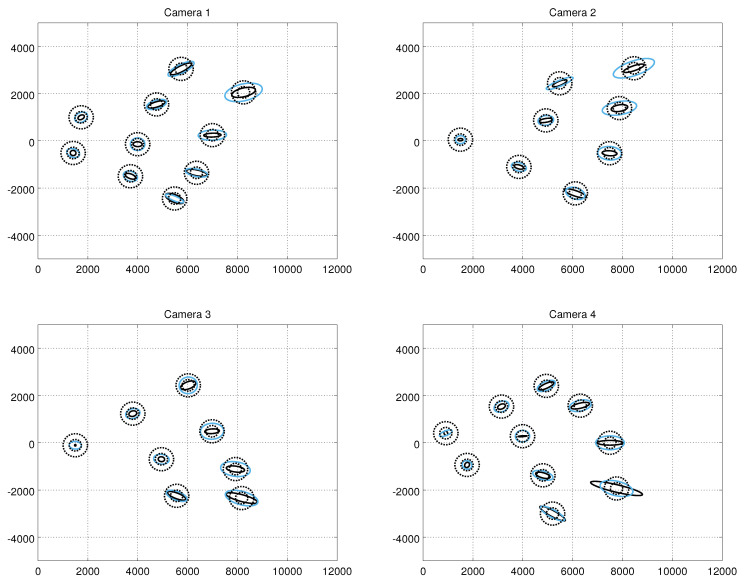
Measurement error ellipses at the confidence level of 95% computed from the human experiments. The solid black ellipses refer to the ground truth, the smaller dashed black circles to the constant noise model with σ = 10, the larger dashed ellipses to the constant noise model with σ = 50, and the blue ellipses to the results obtained with the autocalibration method. The error ellipses are shown in the camera-centric Cartesian plan-view coordinate system.

Figure 7 shows the error covariance ellipses resulting from the ground truth measurements, from the constant noise model (σ = 50) and from our method at the confidence level of 95%. The results are in the Cartesian frame. As expected, the shape of the error ellipses of the true measurement noise depends on the range, and the major axes become longer at high distances. The widths of the ellipses do not vary much. However, there are observable variations between the shapes of the ellipses of different cameras. For example, at the distance of approximately 10 metres, the lengths of the major axes of the ellipses of Camera 1 are approximately two times longer compared to Camera 3. At the Cartesian frame, the shape of the constant noise model’s covariance error ellipse is circular throughout the imaging area, whereas the shape of the error ellipse of our model follows closer to the ground truth. The error covariance ellipses from the human measurements are shown in Figure 8. Visual comparison to the results from the robot measurements suggest that the results are close to similar.

**Figure 9:**
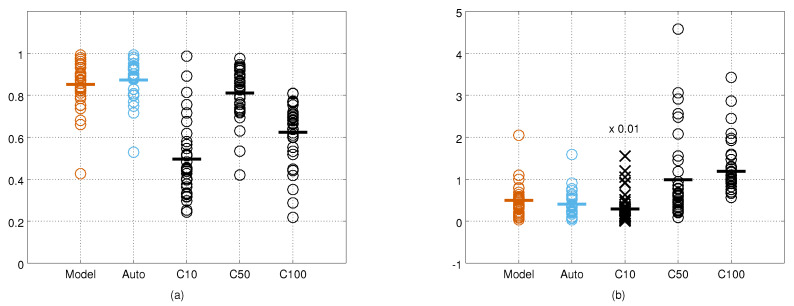
The cross-correlation (**a**) and the Kullback–Leibler divergence (**b**) values between the ground truth error covariance matrices and the estimated covariance matrices computed from the robot measurements. C10 refer to the constant noise model with σ=10 mm, and C50 and C100 to the constant noise models with σ=50 mm and σ=100 mm, respectively. In (**b**), the C10 values (marked with ×) are divided by a factor of 100 for visualisation purposes. Horizontal lines indicate the mean values.

**Figure 10:**
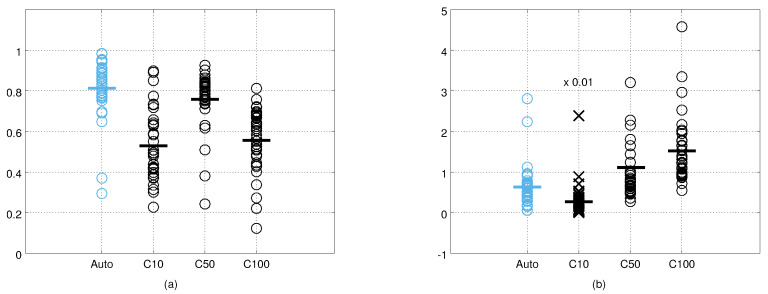
The cross-correlation (**a**) and the Kullback–Leibler divergence (**b**) values between the ground truth error covariance matrices and the estimated covariance matrices computed from human measurements. C10 refer to the constant noise model with σ=10 mm, and C50 and C100 to the constant noise models with σ=50 mm and σ=100 mm, respectively. In (**b**), the C10 values (marked with ×) were divided by a factor of 100 for visualisation purposes. Horizontal lines indicate the mean values.

The cross-correlation and KL divergence values from all the cameras and test points are shown in Figure 9. Visual inspection suggests that the measurement error covariance matrices obtained with the autocalibration method are the most similar to ground truth covariance matrices, and that the covariance matrices of the constant noise model with σ = 10 are the least similar. Using the cross-correlation as the metric for similarity, the constant noise model with σ = 50 performs close to the results obtained with our method. However, using the KL divergence as the error metric, both our methods outperform the constant noise model with σ = 50. The KL divergence values form more compact groups for our methods indicating that our noise model adapts better to the varying measurement angles and distances. The quantitative results for each camera and both metrics are provided in Table 1 and Table 2. The results obtained by the human measurements are shown in Figure 10. The relative performance of different methods are similar to the results obtained from the robot data, but the absolute performance is on average slightly worse.

### 5.2. Tracking

The RMSE values from each tracking experiment are shown in Table 3, Table 4 and Table 5. Constant noise models with σ=10 and σ=50 clearly outperform our method when using the *q* value of 10. However, when using the *q* value of 100 or 1000, our method gives more than 5% better accuracy in 19 out of 36 cases, and 1% or better accuracy in 25 cases. Compared to the constant noise model with σ=100, our method gives 5% or more accurate results in 16 out of 27 cases (all *q* values), and performs as good or better in 25 cases.

**Figure 11:**
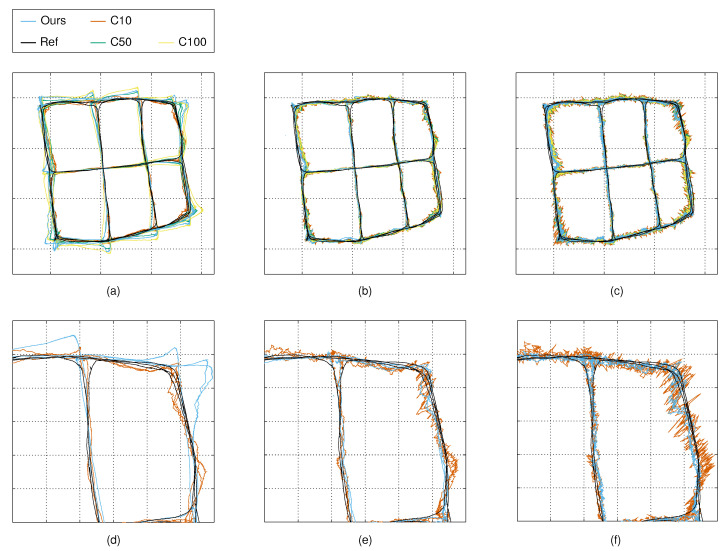
Example trajectories from tracking experiment 1: images (**a**–**c**) show the estimated trajectories using the *q* values of 10, 100 and 1000; images (**d**–**f**) show the zoomed-in trajectories obtained with our noise model, and with the constant noise model with σ=10. C10 refers to the constant noise model with σ=10 mm, and C50 and C100 refer to the constant noise models with σ=50 mm and σ=100 mm, respectively. In (**a**–**c**), the tic marks are at two-metre intervals; and in (**d**–**f**) these are at 0.5 m intervals.

Figure 11 shows that with a small *q* value, the tracker does not give as much weight to the observations, and the changes in the system state are relatively slow. The phenomenon is clearly noticeable in the sharp turns that the person makes at the grid points. The constant noise model with σ=10 makes an exception, and the position estimates of the tracker follow the reference trajectory relatively well. As the *q* value is increased, the trackers using our measurement noise model and the constant measurement noise models with σ=50 and σ=100 start to follow the reference trajectories more closely. However, the tracker with the constant measurement noise model with σ=10 starts to follow the noisy observations which results in the higher variance of the position estimate.

## 6. Discussion and Conclusions

Depth cameras generally suffer from range measurement noise. Typically, the noise depends on the target being observed, and especially on its distance and direction relative to the sensor. The accurate measurement of noise estimates optimally weights the observations, and gives more importance to the measurements with higher confidence. In applications such as people detection and tracking, this measurement noise should be modelled for more precise trajectory estimation, and data association and fusion in multi-sensor systems. We presented an approach for modelling the measurement noise for the plan-view-based people tracking applications. In our approach, we treat the observations in a camera-centric polar coordinate system parallel to the floor plane. Both bearing and range measurement noise values are dependent on the position of the observed target relative to the sensor. In our implementation, we model the functions with two variance maps, one for the range measurement noise and one for the angle measurement noise.

We compared the estimated noise covariance matrices to the true measurement noise captured from static targets. Additionally, we compared our noise model to the constant measurement noise model typically used in people tracking applications. For comparison metrics, we used the cross-correlation and the Kullback–Leibler divergence values. The results show that our approach outperforms the constant noise model in both metrics. The constant noise model is sensitive to the selected value, and it does not adapt to the target’s state.

We evaluated the performance of the proposed measurement noise model with our Kalman filter-based people tracker system as well. The filter uses a (nearly) constant velocity state-space model, and the allowed rate of the changes in the velocity depends on the magnitude of the process noise covariance, defined by the *q* value of Equation (3). We conducted experiments with *q* values of 10, 100 and 1000. With the low values, the tracker weights less the measurements, and trusts more the dynamic model. Thus, with our measurement noise model, the tracker produces trajectories that lag behind the reference and overshoot when the target performs relatively fast changes in terms of velocity. The constant measurement noise model with low variance (σ=10) gives excessive weight to the (noisy) measurements, and the output of the tracker follows the reference with higher accuracy. However, such a measurement noise model does not follow the real error distribution which may cause non-optimal performance in data association and gating tasks. In this paper, we had only one target being tracked, and we did not limit the search space for the data association. The comparison of different noise models in those tasks are left for future investigations.

As we increased the process noise magnitude, the constant measurement noise model with σ=10 started to follow the noisy measurements, and the result started to strongly deviate around the reference. However, our approach, and the constant measurement noise models with higher variances, produce more stable and accurate results. On average, using *q* values of 100 and 1000, our method delivers as good or better accuracy than the other measurement noise models we tested. In real-life scenarios, the people being tracked may perform rapid manoeuvres, and a relatively high intensity shall be used for the process noise covariance.

The measurement noise of the depth cameras depends on various factors, which can be completely unknown, or difficult to model in practise. Our approach provides a relatively simple and practical way for determining the measurement noise model for depth camera-based people tracking applications. It is especially beneficial in the autocalibration context, where we assume that the camera network is constructed ad hoc, and any pre-calibration steps should be avoided. Additionally, the method adapts to the sensors with different measurement noise characteristics. Our approach does not model random errors that originate, e.g., from low NIR reflectivity materials or multi-path effects. To model such errors, one possible approach could be to include contextual information to the system. For example, with a more advanced object detection and classification approach it could be possible to determine object-specific measurement noise models.

Our approach operates in plan-view domain, and using the highest points of the persons as observations, tracks the people in 2D floor plane coordinate system. However, the suggested method is not only limited to 2D, and could be extended to 3D tracking as well. For example, a skeleton detection algorithm cloud be used for detecting body joints from the depth camera images, and the variance maps could be generated as 3D lookup-tables. In our experiments, the measurements used to construct the variance maps were sparsely and unevenly distributed on the floor-plane coordinate system. In such a scenario, the interpolation method and associated hyperparameters may have a significant effect on the results. Extending the approach to 3D, and experimenting with a wider set of interpolation methods and parameter values are plans for future work.

## Figures and Tables

**Table 1:** The mean cross-correlation values and the standard deviations (in parenthesis) between the ground truth error covariance matrices and the estimated error covariance matrices from each camera. The top five rows correspond to the robot measurements, and the bottom four rows correspond to the human measurements.

	Camera 1	Camera 2	Camera 3	Camera 4	All
Model-based	0.88 (0.07)	0.86 (0.07)	0.82 (0.09)	0.82 (0.17)	0.85 (0.11)
Auto	0.91 (0.06)	0.88 (0.06)	0.87 (0.06)	0.83 (0.14)	0.87 (0.09)
σ=10	0.45 (0.16)	0.49 (0.15)	0.50 (0.15)	0.52 (0.25)	0.49 (0.18)
σ=50	0.84 (0.09)	0.83 (0.07)	0.86 (0.11)	0.75 (0.17)	0.81 (0.12)
σ=100	0.65 (0.11)	0.61 (0.11)	0.63 (0.14)	0.59 (0.20)	0.62 (0.15)
Auto	0.87 (0.09)	0.79 (0.09)	0.76 (0.20)	0.80 (0.17)	0.81 (0.14)
σ=10	0.51 (0.15)	0.54 (0.17)	0.54 (0.17)	0.52 (0.20)	0.53 (0.17)
σ=50	0.82 (0.05)	0.77 (0.11)	0.76 (0.21)	0.71 (0.14)	0.76 (0.14)
σ=100	0.60 (0.13)	0.56 (0.14)	0.56 (0.20)	0.53 (0.16)	0.56 (0.15)

**Table 2:** The mean Kullback–Leibler divergence values and the standard deviations (in parenthesis) between the ground truth error covariance matrices and the estimated error covariance matrices from each camera. The top five rows correspond to the robot measurements, and the four bottom rows to the human measurements.

	Camera 1	Camera 2	Camera 3	Camera 4	All
Model-based	0.39 (0.23)	0.46 (0.21)	0.56 (0.29)	0.58 (0.59)	0.49 (0.37)
Auto	0.31 (0.19)	0.38 (0.19)	0.41 (0.19)	0.53 (0.45)	0.41 (0.29)
σ=10	43.9 (55.4)	28.4 (30.4)	19.3 (11.5)	37.9 (37.4)	33.2 (37.6)
σ=50	1.21 (1.55)	0.83 (0.78)	0.50 (0.32)	1.32 (0.93)	1.00 (1.03)
σ=100	1.17 (0.42)	1.25 (0.44)	1.20 (0.58)	1.47 (0.96)	1.28 (0.64)
Auto	0.43 (0.25)	0.65 (0.28)	0.83 (0.84)	0.66 (0.61)	0.63 (0.54)
σ=10	23.7 (18.1)	20.4 (14.6)	24.2 (28.4)	44.3 (72.1)	28.8 (41.5)
σ=50	0.71 (0.17)	0.81 (0.40)	1.09 (1.06)	1.76 (2.17)	1.11 (1.29)
σ=100	1.28 (0.52)	1.49 (0.66)	1.65 (1.25)	1.72 (0.77)	1.53 (0.81)

**Table 3:** RMSE values between the estimated position of the target and the reference points in tracking experiment 1. Green cells indicate the cases where our method outperforms the reference method by 5% or more, and light green by 1% or more. Red cells indicate the cases where the reference method outperforms our method at the same thresholds. Gray cells indicate the cases where the difference is in between ±1%.

	Calibration	Ours	σ=10	σ=50	σ=100
	Model based	125.6	122.1	132.5	156.9
q=10	Auto, robot	107.9	89.6	103.0	129.5
	Auto, person	202.0	103.5	173.6	279.0
	Model	105.6	126.2	122.9	122.3
q=100	Auto, robot	82.5	92.5	89.5	89.4
	Auto, person	101.4	100.4	98.9	103.5
	Model	113.9	139.5	129.0	126.3
q=1000	Auto, robot	94.1	109.7	96.2	92.4
	Auto, person	101.3	115.1	102.8	101.0

**Table 4:** RMSE values between the estimated position of the target and the reference points in tracking experiment 2. Cell colours are as in Table 3.

	Calibration	Ours	σ=10	σ=50	σ=100
	Model based	194.1	134.7	165.6	209.1
q=10	Auto, robot	165.5	106.3	133.8	178.2
	Auto, person	336.6	143.0	259.9	427.2
	Model	134.1	132.8	132.8	135.8
q=100	Auto, robot	109.0	107.6	105.3	107.0
	Auto, person	131.5	132.6	135.9	143.8
	Model	130.9	144.9	135.2	132.9
q=1000	Auto, robot	108.5	122.4	110.7	107.9
	Auto, person	132.1	143.1	133.1	132.0

**Table 5:** RMSE values between the estimated position of the target and the reference points in tracking experiment 3. Cell colors are as in Table 3.

	Calibration	Ours	σ=10	σ=50	σ=100
	Model based	147.0	126.2	140.6	173.7
q=10	Auto, robot	126.0	96.6	116.3	152.7
	Auto, person	254.2	117.6	223.0	369.8
	Model	117.1	131.1	127.8	126.9
q=100	Auto, robot	92.6	99.7	96.7	96.9
	Auto, person	111.5	111.0	111.8	118.0
	Model	124.6	151.4	135.5	131.6
q=1000	Auto, robot	97.7	122.9	104.4	99.9
	Auto, person	110.8	122.9	113.1	111.9

## Data Availability

Not applicable.
